# Supplementary material for: Global LiDAR land elevation data reveal greatest sea-level rise vulnerability in the tropics
Source: Nat Commun. 2021 Jun 29;12:3592. doi: 10.1038/s41467-021-23810-9 (PMC8242013; doi:10.1038/s41467-021-23810-9)
Supplement: Supplementary file 1 — Supplementary Information for “Global LiDAR land elevation data reveal greatest sea-level rise vulnerability in the tropics” by Hooijer and Vernimmen [file 41467_2021_23810_MOESM1_ESM.pdf]

Supplementary Information for "Global LiDAR land elevation data reveal greatest sea-level rise vulnerability in the tropics" by Hooijer and Vernimmen

Supplementary Table 1: Review of land surface subsidence (LSS) rates reported for coastal zones in recent large-scale studies.

**Region**	**Area**	**Soil**	**Method**	**Period**	**Reference**	**Subsidence ($mm\ yr^{-1}$)**	
						Rural	*Urban*
Africa	Nile delta	Mineral	Sentinel-1 satellite data	2015-2019	24	6-20	12-20
Africa	Lagos	Mineral	Geodetic data	2003-2019	25		2-87
East Asia	Beijing-Tianjin-Hebei	Mineral	InSAR satellite data	2012-2018	26		>50
East Asia	Shanghai	Mineral	Ground monitoring	2000-2017	27		5-12
East Asia	Pearl River delta	Mineral	InSAR satellite data	2007-2010	28	~2.5	
East Asia	Yellow River delta	Mineral	Model	Projection	29	>5	
East Asia	South Korea	Mineral	InSAR satellite data	2015-2017	30	187	
Europe	Netherlands	Mixed	Model	Projection	31	3-8	
Greater Southeast Asia	Ganges-Brahmaputra-Meghna delta	Mineral	Satellite altimetry	1993-2012	21	1.5-7.2	
Greater Southeast Asia	Ganges-Brahmaputra-Meghna delta	Mineral	Review	Varied	32	~5.6	
Greater Southeast Asia	Jakarta & other cities	Mineral	InSAR satellite data	2007-2009	33		42-72
Greater Southeast Asia	Mekong delta	Mineral	Model & InSAR	1990-2015	34	11	~70
North America	Coastal Louisiana	Mineral	Ground monitoring	6-10 years	35	9 ± 1	
North America	Chesapeake Bay	Mineral	Ground monitoring	Since 1940s	36	1.-4.8	
North America	Mississippi delta	Mineral	Ground monitoring	Since 1990	37	~3.4	
North America	Sacramento delta	Mixed	Ground monitoring	1950-1990	38	3.8-4.8	

References

24. Rateb, A. & Abotalib, A. Z. Inferencing the land subsidence in the Nile Delta using Sentinel-1 satellites and GPS between 2015 and 2019. Science of The Total Environment **729**, 138868 (2020).
25. Ikuemonisan, F. E. & Ozebo, V. C. Characterisation and mapping of land subsidence based on geodetic observations in Lagos, Nigeria. Geodesy and Geodynamics **11**, 151–162 (2020).
26. Zhou, C. et al. Land Subsidence Response to Different Land Use Types and Water Resource Utilization in Beijing-Tianjin-Hebei, China. Remote Sensing **12**, 457 (2020).
27. He, X.-C., Yang, T.-L., Shen, S.-L., Xu, Y.-S. & Arulrajah, A. Land Subsidence Control Zone and Policy for the Environmental Protection of Shanghai. IJERPH **16**, 2729 (2019).
28. Wang, H. et al. InSAR reveals coastal subsidence in the Pearl River Delta, China: Coastal subsidence in the PRD. Geophysical Journal International no-no (2012) doi:10.1111/j.1365-246X.2012.05687.x.

29. Changxing, S. et al. Land Subsidence as a Result of Sediment Consolidation in the Yellow River Delta. Journal of Coastal Research **231**, 173–181 (2007).
30. Suresh, K. P. V., Kim, D., Jung, J. & Cho, Y.-K. Application of PS-InSAR Technique for Measuring the Coastal Subsidence in the East Coast of South Korea. in 2019 URSI Asia-Pacific Radio Science Conference (AP-RASC) 1–3 (IEEE, 2019). doi:10.23919/URSIAP-RASC.2019.8738313.
31. Koster, K., Stafleu, J. & Stouthamer, E. Differential subsidence in the urbanised coastal-deltaic plain of the Netherlands. Netherlands Journal of Geosciences **97**, 215–227 (2018).
32. Brown, S. & Nicholls, R. J. Subsidence and human influences in mega deltas: The case of the Ganges–Brahmaputra–Meghna. Science of The Total Environment **527–528**, 362–374 (2015).
33. Chaussard, E., Amelung, F., Abidin, H. & Hong, S.-H. Sinking cities in Indonesia: ALOS PALSAR detects rapid subsidence due to groundwater and gas extraction. Remote Sensing of Environment **128**, 150–161 (2013).
34. Minderhoud, P. S. J. et al. Impacts of 25 years of groundwater extraction on subsidence in the Mekong delta, Vietnam. Environ. Res. Lett. **12**, 064006 (2017).
35. Nienhuis, J. H., Törnqvist, T. E., Jankowski, K. L., Fernandes, A. M. & Keogh, M. E. A New Subsidence Map for Coastal Louisiana. GSAT 60–61 (2017) doi:10.1130/GSATG337GW.1.
36. Eggleston, J. & Pope, J. Land subsidence and relative sea-level rise in the southern Chesapeake Bay region. (2013).
37. Morton, R. A. & Bernier, J. C. Recent Subsidence-Rate Reductions in the Mississippi Delta and Their Geological Implications. Journal of Coastal Research **263**, 555–561 (2010).
38. Mount, J. & Twiss, R. Subsidence, Sea Level Rise, and Seismicity in the Sacramento–San Joaquin Delta. SFEWS **3**, (2005).
