# Supplementary material for: Global LiDAR land elevation data reveal greatest sea-level rise vulnerability in the tropics
Source: Nat Commun. 2021 Jun 29;12:3592. doi: 10.1038/s41467-021-23810-9 (PMC8242013; doi:10.1038/s41467-021-23810-9)
Supplement: Supplementary file 4 — Description of additional supplementary files [file 41467_2021_23810_MOESM4_ESM.docx]

Description of additional supplementary information

Title: Supplementary data

Description: Coastal lowland area and population below 2 and 0 m +MSL, with confidence levels at 68% and 95%. Areas calculated from different GDEMs (SRTM9013, MERIT14, CoastalDEM15 and TanDEM-X38) compared to GLL_DTM_v18 at 0.05-degree resolution, for countries ranked by land area greater than 12,000 km2 below 2 m +MSL. For consistency, numbers are calculated within the SRTM coverage extent, between 60N and 56S. Population data from ref18.
